# Paraneoplastic Vasculitis in a Patient With Breast Cancer at an Unusual Age: A Case Report

**DOI:** 10.1155/crom/6699329

**Published:** 2026-05-22

**Authors:** Angélica Mandujano, Melissa Golubov, Víctor Gutiérrez

**Affiliations:** ^1^ Department of Health Care, Universidad Autónoma Metropolitana-Unidad Xochimilco, Mexico City, Mexico, uam.mx; ^2^ Faculty of Medicine, Universidad Nacional Autónoma de México, Mexico City, Mexico, unam.mx; ^3^ Bachelor′s Program in Medicine, Universidad Autónoma Metropolitana-Unidad Xochimilco, Mexico City, Mexico, uam.mx

**Keywords:** breast cancer, cutaneous vasculitis, leukocytoclastic vasculitis, paraneoplastic syndrome, purpura

## Abstract

We report the case of an 87‐year‐old woman whose initial presentation was palpable purpura. Henoch–Schönlein purpura was initially diagnosed, for which she received intravenous methylprednisolone and epinastine, resulting in complete remission of the skin lesions. After discontinuation of corticosteroid therapy, she experienced multiple relapses over the ensuing months with an incomplete response to medical treatment. Metastatic breast cancer was diagnosed 7 months later, which retrospectively suggested a paraneoplastic etiology for the vasculitis. Although the association between cutaneous vasculitis and malignancy—particularly hematologic cancers—is well documented, the underlying mechanisms remain poorly understood. The link with breast cancer is rare, though sporadically reported. Vasculitis in this context may occur before, during, or after cancer diagnosis or treatment. Notably, only two cases of breast cancer–associated vasculitis have been reported in patients aged 80 or older. Paraneoplastic vasculitis typically resolves with treatment of the underlying malignancy. However, given the presence of metastatic disease in this patient, immunosuppressive therapy with azathioprine was needed, resulting in complete resolution of lesions.

## 1. Introduction

The term *vasculitis* refers to the inflammation of blood vessel walls. The 2012 Revised International Chapel Hill Consensus Conference classifies vasculitides into well‐defined categories; however, they can also be classified according to vessel size, etiology, pathogenesis, clinical manifestations, or other parameters. Systemic vasculitides are a group of disorders (including single‐organ vasculitis, Henoch–Schönlein purpura, polyarteritis nodosa, and microscopic polyangiitis) that may arise as primary diseases or occur secondary to an underlying condition, including cancer [1]. Paraneoplastic vasculitis may occur prior to, concurrently with, or even after cancer [2].

When observed, paraneoplastic vasculitis is most commonly associated with hematologic malignancies and is only rarely described in the context of solid tumors. Few cases of vasculitis related to breast cancer have been published, with particularly limited reports in octogenarian women, for whom systematic screening remains controversial. We present the case of an 87‐year‐old woman who initially presented with purpura and was later found to have metastatic breast cancer. Awareness of the relationship between cancer and vasculitis is necessary to avoid delaying diagnosis and providing specific treatment.

## 2. Case Presentation

An 87‐year‐old woman presented with cutaneous vasculitis as the initial manifestation of breast cancer. She had a family history notable for pancreatic cancer (father), pulmonary thromboembolism (sister), stroke (sister), and three children with hemophilia. Her past medical history included chronic biomass exposure, long‐standing hypertension, dyslipidemia, chronic obstructive pulmonary disease (COPD), atrial fibrillation, and chronic ischemic heart disease and treated with telmisartan 80 mg QD, bisoprolol 2.5 mg QD, atorvastatin 20 mg QD, transdermal glyceryl trinitrate 25 mg QD, and nocturnal oxygen therapy.

**Figure 1:**
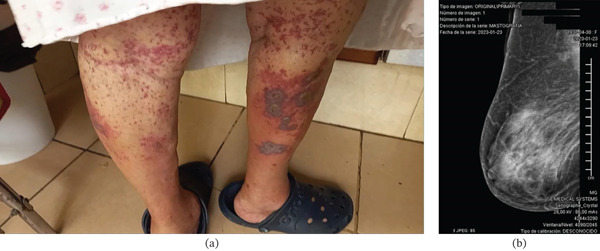
(a) Palpable purpura in lower extremities showing necrotic areas. (b) Mammography showed a high‐density irregular, partially spiculated mass with architectural distortion and fine, amorphous, segmental, and pleomorphic calcifications with diffuse skin thickening on the left breast corresponding with a BI‐RADS 5. Breast ultrasound reported BI‐RADS 4C (not shown).

**Figure 2:**
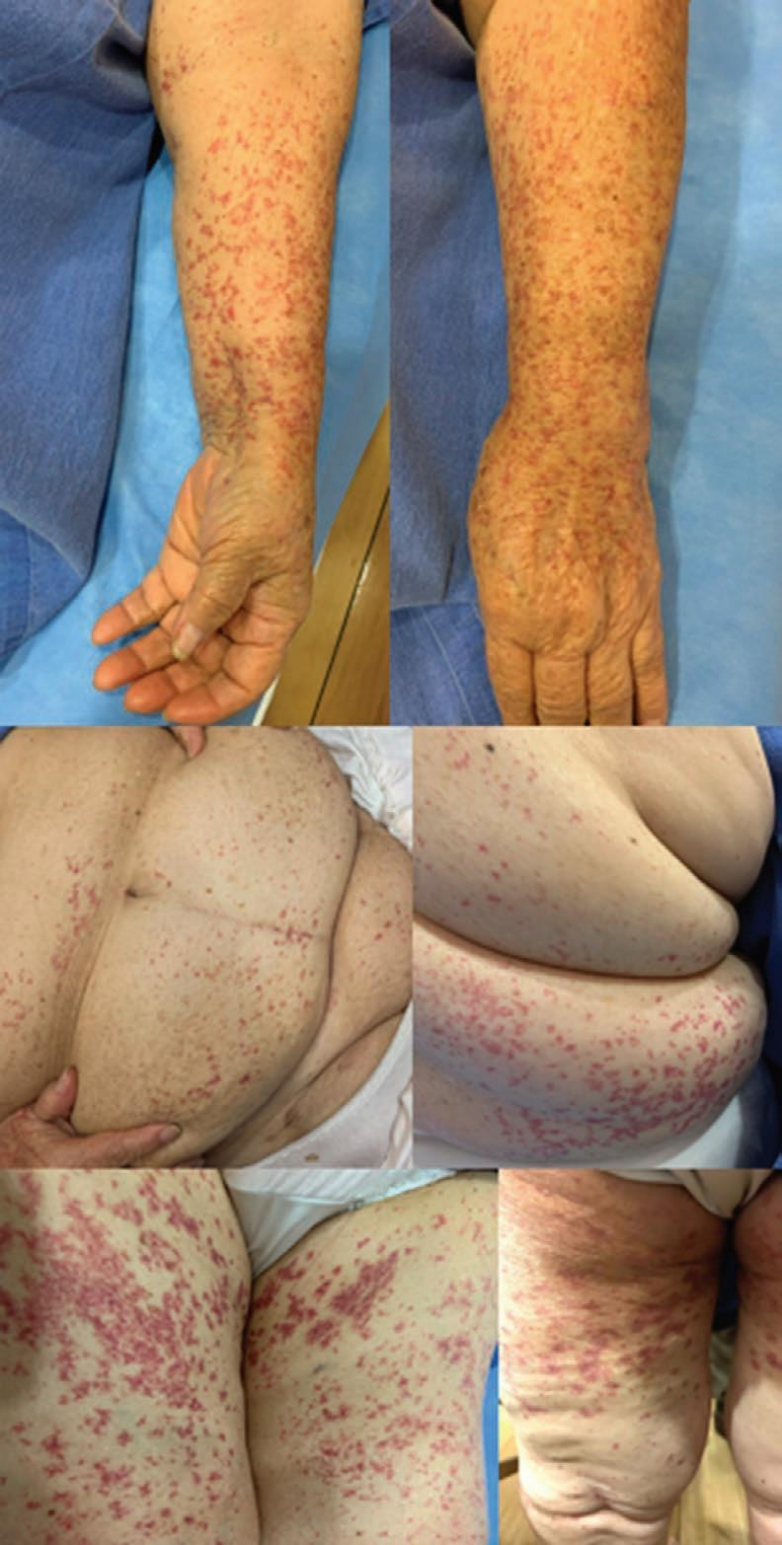
Skin examination revealed a tender nonblanching dermatosis characterized by palpable purpura. Lesions were primarily distributed proximally on both the anterior and posterior aspects of the lower extremities, with additional distal involvement of the upper extremities, with fewer, grouped lesions on the trunk, particularly on the posterior region, sparing the face.

In August 2022, she developed isolated petechiae over her thighs, which progressed over 48 h to involve the entire lower extremity and evolved into palpable purpura with areas of necrosis (Figure 1a). She was admitted to the internal medicine service and received the presumptive diagnosis of HSP. Treatment with intravenous methylprednisolone and epinastine was started, with complete remission of lesions. Upon discharge, she was prescribed oral steroids. Shortly after discontinuation, she reported isolated petechiae on lower extremities that spontaneously resolved within a few days, with similar episodes occurring approximately every month. In January 2023, she was admitted to the hospital due to community‐acquired pneumonia. During her hospitalization, axillary lymphadenopathy and a breast mass were found. Mammography (Figure 1b) and breast ultrasound were performed. In March 2023, a biopsy followed by a radical mastectomy confirmed invasive ductal breast carcinoma, Nottingham Grade 2. A bone scan showed metastatic disease affecting T3, L2, and the second left costal arch. Chest radiography and computer tomography revealed lung metastases. Treatment with letrozole 2.5 mg daily was started. On December 12, 2023, she again presented with a purpuric rash affecting the lower extremities. Her internist started epinastine, with a poor clinical response. Over the following 2 weeks, the lesions gradually spread to the trunk and upper extremities. Deflazacort 30 mg QD was prescribed, but her symptoms failed to improve, prompting referral to rheumatology. At her initial rheumatology evaluation with our service on January 25, 2025, she exhibited new skin lesions despite ongoing glucocorticoid treatment. Vital signs were as follows: BP 140/80, HR 85 beats/min, RR 16 breaths/min, and oxygen saturation 86%. Physical examination was notable for irregular heart sounds with a systolic murmur and isolated rales in the subscapular regions. Musculoskeletal examination showed Heberden and Bouchard nodes in both hands, bilateral rhizarthrosis, and gonarthrosis with mild synovial effusion. Skin examination findings are shown in Figure 2. Laboratory evaluation revealed a white blood cell count of 14.4 × 10^9^/L, a hemoglobin of 15 g/dL, and a platelet count of 249 × 10^9^/L. Serum glucose was 84 mg/dL, blood urea nitrogen was 42 mg/dL, and creatinine was 1.42 mg/dL. Electrolytes, creatine kinase, liver, lipid, and thyroid function panels were unremarkable. C‐reactive protein was 6.14 mg/L, and erythrocyte sedimentation rate was 23 mm/h. Coagulation studies showed a prothrombin time of 12.4 s and an activated partial thromboplastin time of 37.5 s. Urine sediment analysis showed no evidence of leukocyturia, hematuria, proteinuria, casts, or hemoglobin. Urine culture was negative. Spot urine protein‐to‐creatinine and microalbumin‐to‐creatinine ratios were normal. The fecal occult blood test was negative. Immunoglobulin levels, C3, and C4 were within normal ranges. Antinuclear antibodies were positive at a titer of 1:160 with a speckled pattern on HEp‐2 cells (IIFA). The following serologies were all negative: rheumatoid factor, anti‐dsDNA (CLIFT), lupus anticoagulant (DRVVT), IgM, IgG, and IgA anticardiolipin and anti–beta 2 glycoprotein antibodies, HIV, HBV, HCV, antineutrophil cytoplasmic antibodies, antimyeloperoxidase antibodies, anti–Proteinase 3 antibodies, and cryoglobulins.

**Figure 3:**
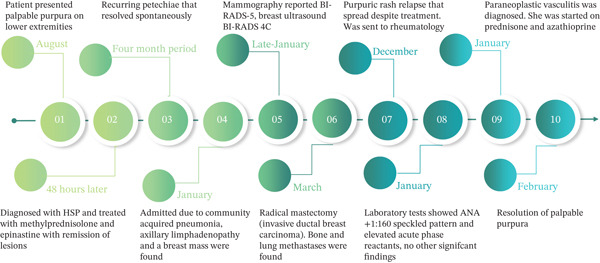
Timeline summarizing the patient′s clinical manifestations, treatment, and disease course. The figure illustrates the temporal relationship between a cutaneous vasculitis and breast cancer, demonstrating an asynchronous pattern in which vasculitis preceded the cancer diagnosis. The time intervals shown are approximate and highlight the most relevant events. Numbers within circles indicate the sequence of events. Note that the time elapsed between the diagnosis of vasculitis and the identification of a palpable breast mass was approximately 5 months and a definitive diagnosis was made 2 months later.

Given the negative laboratory workup, the clinical history, and the onset of cutaneous vasculitis months prior to the diagnosis of metastatic cancer, together with a relapsing course, paraneoplastic vasculitis was considered the most likely diagnosis. A skin biopsy was offered; however, the patient declined after discussion of potential risks related to frailty, comorbidities, and the need for immunosuppressive therapy. The expected benefit was considered limited, given the typical clinical presentation of small‐vessel vasculitis, the possibility of nonspecific findings, and the minimal anticipated impact on management. Treatment was initiated with prednisone at an initial dose of 50 mg QD (~0.5 mg/kg) for 4 weeks, followed by 10 mg decrements every 10 days to 20 mg, and subsequently 2.5 mg decrements every 10 days until discontinuation. Azathioprine was started at 75 mg daily, resulting in resolution of the palpable purpura and sustained remission without relapse. A timeline of the evolution from diagnosis to remission is shown in Figure 3.

## 3. Discussion

According to the size of the vessel predominantly affected, systemic vasculitis can be classified as small‐vessel (e.g., IgA vasculitis and ANCA‐associated vasculitis), medium‐vessel (e.g., Kawasaki disease and polyarteritis nodosa), large‐vessel (e.g., Takayasu arteritis and giant cell arteritis), or variable‐sized (e.g., Behcet disease) vasculitis [3, 4]. Other categories in the nomenclature include single‐organ vasculitis, which encompass cutaneous leukocytoclastic vasculitis, as well as vasculitides associated with a likely etiology, including cancer [1].

Cutaneous vasculitis is a heterogeneous entity and may be confined to the skin or represent a manifestation of systemic vasculitis [5]. Up to 55% of cases are idiopathic, while 15%–20% are associated with infections, 15%–20% with autoimmune diseases, 10%–15% with drugs, and around 5% with malignancy [6]. Most cases are self‐limited and resolve within 3–4 weeks. However, further diagnostic workup is warranted in recurrent or chronic presentations [5]. Cutaneous vasculitis has been reported in association with a wide range of solid tumors, including lung, colon, renal, prostate, breast, head, and neck cancers, as well as hematologic malignancies [7, 8], with a higher frequency in the latter [2, 9]. The main clinical sign is typically palpable purpura of the lower extremities. Similar to previous reports, our case had bilateral, somewhat symmetrical skin lesions predominantly in the lower extremities without involvement of the face [10].

The reported overall prevalence of vasculitis is around ~2%–5% [2, 7, 11, 12], with small‐vessel cutaneous vasculitis being the most common subtype [2, 13]. However, epidemiologic estimates remain challenging due to the lack of standardized classification criteria, geographic variability, and limited registry data [14].

Several studies have evaluated the association between cutaneous vasculitis and malignancy. Podjasek et al. identified solid tumors in 17 of 812 cases of cutaneous vasculitis within 12 months of the onset of vasculitis. Of these, 18% were related to breast cancer, and vasculitis preceded the diagnosis of malignancy in all cases. The authors reviewed published cases of paraneoplastic vasculitis until March 2010 and found 53 cases of cutaneous vasculitis associated with solid organ malignancies, which they later compared to their cases. Of note, five breast carcinomas were found: One was synchronous to the vasculitis, data were missing for two cases, and a long period elapsed between the cancer diagnosis and the onset of cutaneous vasculitis in the remaining two cases, making an association unlikely [7]. In a similar study conducted over a 15‐year period, among 596 patients with vasculitis, 15 were diagnosed with a solid tumor within 12 months. Only one involved breast cancer; however, the vasculitis in that case was giant cell arteritis [13]. Loricera et al. reported a 20‐year series of 766 cases of cutaneous vasculitis. Of the 421 adults, 3.8% were found to have an underlying malignancy within 12 months of the onset of vasculitis; seven were solid tumors, and only two were associated with breast cancer [15]. In an 18.5‐year retrospective review, Hutson and Hoffman identified 12 cases in which cancer was diagnosed within 12 months of vasculitis onset. Solid tumors accounted for 50% of malignancies, and cutaneous leukocytoclastic vasculitis was confirmed histologically in four patients. No cases involved breast cancer [16]. In another 10‐year series, 8 of 192 patients with cutaneous vasculitis were diagnosed with malignancy. Only two were solid tumors, and neither case was associated with breast cancer [11].

In a retrospective study, Fain et al. analyzed 60 patients with malignancy‐associated vasculitis. The most frequent subtype was cutaneous leukocytoclastic vasculitis (45%), followed by polyarteritis nodosa. In a minority of cases, the diagnosis of vasculitis preceded that of the malignancy: In 14 patients, vasculitis occurred 3.8–24.6 months before cancer was identified. Of the 24 solid tumors reported, only two were associated with breast cancer [2]. These findings suggest that although the association between breast cancer and cutaneous vasculitis has been described, it remains rare. This case is particularly unusual, considering that of all cases in the aforementioned reports, only two occurred at Age 80 or older. In one, due to the long time between diagnoses, it cannot be directly attributed to cancer, and in the other, the information is not available [7]. A relationship between HSP and solid tumors has also been described. In a retrospective study of 103 patients with HSP, 23 were associated with malignancy; none involved breast cancer. Nonetheless, isolated case reports can be found [17]. This patient was diagnosed by another physician with HSP; however, our patient did not exhibit extracutaneous involvement, making the diagnosis of HSP unlikely. Conceptually, cutaneous vasculitis may represent a limited expression of systemic vasculitis [1]. However, in elderly patients and those with comorbidities, systemic manifestations can be misleading and may reflect underlying conditions rather than systemic vasculitis. In this patient, advanced gonarthrosis could have been misinterpreted as arthritis related to HSP. Renal impairment could be explained by cardiac and COPD decompensation. Importantly, there was no evidence of glomerulonephritis, either clinically or on urinary sediment analysis, and no proteinuria was detected. Therefore, a thorough clinical evaluation is necessary to avoid misdiagnosis in this population.

The relationship between vasculitis and malignancy can be classified into three categories: vasculitis as a true paraneoplastic syndrome, malignancies mimicking vasculitis, and vasculitis mimicking malignancies. In true paraneoplastic vasculitis, there is typically a temporal relationship between both diagnoses, a parallel clinical course, and improvement or resolution with cancer treatment. Malignancies may mimic vasculitis due to vascular invasion, an example being angiocentric T‐cell lymphoma. The last category includes patients with a mass suggestive of malignancy, but finally, after exhaustive investigation, vasculitis is diagnosed [18]. In our case, the presence of metastatic breast cancer is well established. Although a skin biopsy could not be performed, histopathological findings in malignancy‐associated vasculitis most commonly correspond to leukocytoclastic vasculitis [19], reflecting true vasculitic inflammation rather than neoplastic infiltration. Biopsy findings of cutaneous vasculitis in patients with solid tumors can include vasculitis in superficial dermal vessels with fibrinoid necrosis, endothelial cell swelling, and vessel wall infiltrates composed of neutrophils, leukocytoclasia, and erythrocyte extravasation. Direct immunofluorescence reveals deposits of immune complexes, mainly IgM, IgG, and complement (C3) [20].

Considering the clinical presentation, along with the patient′s favorable response to glucocorticoids and immunosuppressive therapy, the findings are consistent with a paraneoplastic syndrome. The initial recurrence of symptoms can be explained by the persistence of metastatic disease, which could not be eradicated; however, the vasculitis has remained controlled with immunosuppressive treatment without the need for continued glucocorticoids. It is important to emphasize that, given the low frequency of cancer‐associated vasculitis, the diagnosis is primarily based on the temporal relationship between vasculitis and the underlying malignancy, clinical features, and response to oncologic therapy. To date, no specific diagnostic criteria or definitive tests are available to establish this diagnosis. Even skin biopsy findings, typically showing leukocytoclastic vasculitis, may be seen in a wide range of conditions. Vasculitis has also been associated with breast cancer treatment. In some patients, distinguishing between paraneoplastic and drug‐induced vasculitides can be challenging. Proposed mechanisms include Type III hypersensitivity reactions due to drug–protein complexes that induce a humoral response leading to immune complex formation, subsequent complement activation, and neutrophil‐mediated vascular injury [21–23]. Reported agents include aromatase inhibitors, selective estrogen receptor modulators (e.g., tamoxifen), and growth factor receptor inhibitors [19, 20, 24]. Other treatments such as intrahepatic chemotherapy, radiotherapy, and bone marrow transplant can also cause vasculitis [20]. In this case, a drug‐related etiology is unlikely as the onset of vasculitis preceded the cancer diagnosis and the initiation of chemotherapy.

Although the causal relationship between vasculitis and malignancy remains incompletely understood, several mechanisms have been suggested. Immune complex‐mediated vascular injury is among the most widely recognized, with tumor antigens potentially participating in complex formation. Cross‐reactivity between antibodies directed against tumor antigens and endothelial cells contributes to vessel wall inflammation, alongside cytokine release through Fc receptor–mediated pathways. Impaired clearance of circulating immune complexes could enhance their deposition within affected vessels. Similar mechanisms underlie IgA‐mediated small‐vessel involvement observed in HSP. Neoplastic cryoglobulinemia, more frequent in hematologic malignancies, has also been associated with cutaneous vasculitic features.

Infectious triggers represent an additional consideration, as certain oncogenic viruses may induce immune complex formation or increase susceptibility to infections in cancer patients, thereby contributing to vascular injury. Finally, the formation of tumor emboli with subsequent vascular occlusion has been proposed as a less common mechanism of cutaneous ischemic injury [9, 15, 18–20, 25–27]. Dermatologic manifestations, in particular, may arise from tumor‐derived growth factors, cytokines, or neurohormones that induce endothelial injury and increase vascular permeability, ultimately leading to inflammation and fibrosis [19, 25]. Cutaneous manifestations may represent the first sign of an underlying malignancy in approximately 1% of cases. Cutaneous vasculitis typically affects small vessels and is often driven by immune complex deposition with subsequent complement activation and vascular damage. Impaired fibrinolysis has also been implicated, especially in cases presenting with palpable purpura, where reduced endothelial release of tissue plasminogen activator and increased levels of its inhibitor promote microthrombus formation and skin necrosis [25].

Other paraneoplastic rheumatic syndromes associated with breast cancer have been described including polymyalgia rheumatica and adult‐onset Still′s disease, highlighting how systemic immune phenomena may precede or accompany cancer progression [28, 29].

As with other paraneoplastic syndromes, the treatment of the underlying malignancy is generally associated with remission of cutaneous manifestations. Treatment depends on the severity of skin or systemic involvement. Systemic corticosteroids remain the cornerstone of therapy, with additional options including nonsteroidal anti‐inflammatory drugs, antimalarials, fibrinolytic agents, intravenous immunoglobulin, biologic therapies, and immunosuppressive agents such as cyclophosphamide, methotrexate, Cyclosporine A, and azathioprine [25]. Treatment recommendations are largely based on case reports and expert opinion [30]. Prognosis is mainly related to that of the underlying neoplasm [2, 9, 15, 25].

In this case, the patient presented with relapsing cutaneous vasculitis in the setting of metastatic breast cancer. As the underlying malignancy could not be eradicated and the patient had multiple comorbidities, the use of glucocorticoids posed an increased risk of complications, particularly metabolic and cardiovascular events. Azathioprine, being among the first‐line treatments [30], was initiated as both an immunosuppressive and a glucocorticoid‐sparing agent. The absence of life‐threatening and extracutaneous involvement, along with multiple comorbidities and infection risk, supported the avoidance of other treatments such as cyclophosphamide or rituximab. Other noteworthy advantages of azathioprine include its oral administration, cost, and availability. The patient achieved sustained remission of vasculitis without glucocorticoids, while metastatic disease remained controlled, supporting the diagnosis of paraneoplastic vasculitis.

Although there is controversy regarding the benefit of breast cancer screening in women over 80 years of age [31, 32], this case highlights the importance of knowing that vasculitis may be related to malignancies, especially in the elderly, and must be considered in the differential diagnosis.

## 4. Conclusion

The relationship between cancer and vasculitis has been described in multiple reports and case series, although the pathophysiological mechanisms have not been completely identified. Breast cancer is rarely associated with vasculitis, but, in older adults, it must be considered among the differential diagnoses. A thorough clinical history and focused evaluation are essential for establishing a definitive diagnosis. While management primarily focuses on treating the underlying malignancy, specific therapies such as glucocorticoids and immunosuppressants may also be employed. The overall prognosis largely depends on the nature and course of the associated neoplasm.

## Funding

No funding was received for this manuscript.

## Ethics Statement

Written informed consent for publication of the clinical case and images was obtained and signed by a family member (daughter).

## Conflicts of Interest

The authors declare no conflicts of interest.

## Data Availability

The data that support the findings of this study are available from the corresponding author upon reasonable request.
